# Evaluating the impact of a UK recovery college on mental well-being: pre- and post-intervention study

**DOI:** 10.1192/bjo.2023.646

**Published:** 2024-04-18

**Authors:** Jon Allard, Adam Pollard, Richard Laugharne, Jamie Coates, Julia Wildfire-Roberts, Michelle Millward, Rohit Shankar

**Affiliations:** Research Team, Cornwall Partnership NHS Foundation Trust, Bodmin, UK; and Cornwall Intellectual Disability Equitable Research (CIDER), University of Plymouth Peninsula School of Medicine, UK; Research Department, Royal Cornwall Hospital NHS Trust, Truro, UK; Recovery College Cornwall, Pentreath Ltd, Truro, UK

**Keywords:** Education and training, psychosocial interventions, social functioning, patients, complimentary therapies

## Abstract

**Background:**

Recovery colleges provide personalised educational mental health support for people who self-refer. The research evidence supporting them is growing, with key components and the positive experiences of attendees reported. However, the quantitative outcome evidence and impact on economic outcomes is limited.

**Aims:**

To evaluate the impact of attending a UK recovery college for students who receive a full educational intervention.

**Method:**

This is a pre- and post-intervention study, with predominantly quantitative methods. Participants recruited over an 18-month period (01.2020–07.2021) completed self-reported well-being (Short Warwick–Edinburgh Mental Wellbeing Scale (SWEMWBS)) and recovery (Process of Recovery (QPR)) surveys, and provided details and evidence of employment and educational status. Descriptive statistics for baseline data and Shapiro–Wilk, Wilcoxon signed-rank and paired *t*-tests were used to compare pre- and post-intervention scores, with Hedges’ *g*-statistic as a measure of effect size. Medical records were reviewed and a brief qualitative assessment of changes reported by students was conducted.

**Results:**

Of 101 student research participants, 84 completed the intervention. Well-being (mean SWEMWBS scores 17.3 and 21.9; *n* = 80) and recovery (mean QPR scores 27.2 and 38.8; *n* = 75) improved significantly (*P* < 0.001; Hedges’ *g* of 1.08 and 1.03). The number of economically inactive students reduced from 53 (69%) to 19 (24.4%). No research participants were referred for specialist mental health support while students. ‘Within-self’ and ‘practical’ changes were described by students following the intervention.

**Conclusions:**

Findings detail the largest self-reported pre–post data-set for students attending a recovery college, and the first data detailing outcomes of remote delivery of a recovery college.

One in six people in England report experiencing mental health problems.^1^ The estimated social and economic cost of poor mental health had risen in 2020 to £119 billion per year.^2^ Awareness of the prevalence and cost of poor mental health has focused policy attention and commitment not only on prevention, but also mental health recovery and recovery values.^3^ Acknowledgment that recovery requires a conceptual and practical shift in focus, from traditional emphasis on symptom remission and medical management toward personalised recovery around individual needs and goals, has gained theoretical recognition and policy support.^4^ Support for mental health systems which reflect such focus has also grown, along with understanding around the importance of recovery-orientated educational approaches.^5^

Recovery colleges have developed in response to this need for a new form of personalised educational mental health support, and have increased in prominence as they have emerged to complement rather than replace specialist mental health services or mainstream educational colleges.^6^ They look to develop a different relationship between services and local communities, utilising interpersonal support and the social and economic factors of mental health in particular, with individuals and communities drawing on their own resources to support recovery journeys.^7,8^ Recovery colleges are founded on adult educational principles,^9^ and focus on the development of shared understanding and education around the rebuilding of lives through personalised recovery journeys.^7^ There is a strong focus on helping students achieve recovery goals through social community and employment opportunities outside of traditional mental health services.^10^ Crucially, emphasis is not only placed on the expertise of trained educational and mental health professionals or facilitators, but the mental health experiences of individuals who attend, with previous students helping not only develop but also deliver group courses.^5^

## The components of recovery colleges

Along with co-production and co-delivery, there are a number of evidence based founding principles and key components of recovery colleges.^11^ Inclusivity, personalisation around course choice and specific delivery methods have been highlighted in review work undertaken through the organisation Implementing Recovery through Organisational Change in the UK,^7,8^ whereas the development of a more recent multi-sourced fidelity model highlights key ‘non-modifiable’ components, including self-referral, co-production, co-delivery and personal tuition support.^9^

This UK fidelity work also highlighted key ‘modifiable’ components of recovery colleges, including location of delivery, explicit focus or distinctiveness of courses and minimal but varied restrictions on accessibility.^9^ Regional funding and operational structures are key drivers for much of this variation,^7,9^ and focus on co-production means recovery colleges evolve in ways that are unique to their local communities and its attendees.^7^ Initially developed in the USA in the 1990s, recovery colleges have been established across at least 20 other countries.^7^ They are most prominent in the UK, where there are at least 80.^12^ Those formally delivering recovery college interventions often include staff from a combination of specialist mental health services, voluntary mental health providers and educational services,^7^ with 64% of recovery colleges employing specialist external staff.^12^ In practice, it is typically a mix of trained mental health workers, such as nurses and support workers, working with specialist professional trainers and those with personal experience of mental health challenges.^8^

## The current impact of recovery colleges

Despite some variation in recovery college models, the evidence base supporting them is growing. Qualitative methods and retrospective surveys are particularly prominent and have heightened understanding of students’ views and opinions during and following attendance, the key components that drive their popularity and the impact they have at the ‘individual’ level.^13^ They have been found to be popular with those attending them, and the staff who work in them.^7^ Students also report increased knowledge and skills around mental health recovery, along with insight and progress toward personal recovery goals.^11,14^

Other key indicators of recovery, such as improvement in hope,^10^ a sense of control and agency,^14^ empowerment,^15^ self-esteem and confidence,^9^ have been reported. A recent thematic synthesis of qualitative research highlights the importance of promoting inclusivity and empowering cultures across recovery colleges,^16^ with the valued impact that comes from peer support from fellow learners also being a key strength.^17^

## The impact of recovery colleges on heath economics

Recent studies have focused on quantifying the financial impact on healthcare services through the analysis of specialist mental health service use data.^18,19^ Reduction in service use and concurrent cost savings for students who have attended recovery colleges have been identified in both the UK^10,19^ and Australia.^18^ Other research work has also identified projected savings for National Health Service (NHS) staff where a UK recovery college operates.^20^

## Current gaps in evidence base for recovery colleges

A detailed comprehensive literature review of 31 original research publications, and another recent commentary, point to the limited rigorous quantitative outcome evidence evaluating the impact of recovery colleges on those who attend.^4,13^ The 2020 literature review highlights five studies detailing quantitative survey data showing significant improvements in wider goals of self-reported well-being, quality of life and the process of recovery.^13^ However, pre- and post-intervention scores are limited because of a focus on small numbers of students (*n* = 32;^11^ *n* = 3^21^), or uniqueness of interventions.^22^ Where data-sets are larger, findings relate to attendance on specific recovery college courses or a specific time frame of attendance at a recovery college, rather than pre- and post-intervention scores following a full recovery college intervention.

Specific economic outcomes around factors such as employment status pre- and post-intervention are also lacking.^4^ Evidence around social contacts, social inclusion and employment opportunity have been cited following recovery college intervention, but where specific employment outcomes have been studied, findings are limited and inconclusive.^10,11^

This study reports data from Recovery College Cornwall (RCC), the first recovery college in Cornwall, a rural area in the south-west of the UK (population of 538 000). RCC and the specific intervention provided for those included in this study are detailed in Appendix 1.

The purpose of this pre- and post-intervention study is to evaluate the impact of RCC on those who attended and received the full intervention, with specific focus on the following research questions:

- Do students’ self-reported outcome measures of well-being and recovery change following the RCC intervention?
- Does employment or educational status change for students following the RCC intervention?
- Are students receiving the RCC intervention supported by, referred into or discharged from specialist mental health NHS services?

Findings are intended to also help address the identified gaps in the current evidence base across recovery colleges outlined above.

A brief qualitative assessment of the ‘changes’ described by students at exit (post-RCC intervention) was also undertaken.

## Method

The Strengthening the Reporting of Observational Studies in Epidemiology (STROBE) checklist for cohort studies was used to guide and report on the project.

### Ethics

UK NHS ethics and Health Regulatory Authority approval was obtained for this study (19/YH/0411, Yorkshire & The Humber – Leeds East Research Ethics Committee; IRAS project identifier 269687). Students were offered the opportunity to consent to take part in all (or some) of the research measures detailed below. Any data presented is with participants’ explicit written consent.

### Participants

All students enrolling at RCC between January 2020 and July 2021 were eligible to take part in the research study running alongside RCC. Enrolment to RCC involved potential students meeting with a learning support worker (LSW), completing enrolment documentation and providing certain documentation, such proof of identity, residence and employment status. Following enrolment and before commencement of RCC courses, students were approached either by the study researcher or their LSW about the research study.

When asked to consent to take part in the study, students were also asked to consent to specific data (collected during their enrolment and at exit) to be used as part of the research study. The components of these data are detailed below. They were also asked to consent to a researcher accessing and collecting data from their NHS medical records, and to complete well-being and recovery surveys. Research participants receiving the full intervention are defined as RCC students who were enrolled, completed courses and received LSW support (detailed in Appendix 1) and were then formally exited (an agreed discharge also referred to as graduation).

### Data sources

#### Baseline data

Demographic data were collected at enrolment to RCC, with students consenting for its use in the research study.

#### Surveys

Surveys were completed during enrolment to RCC and before commencement of RCC courses, and following exit from RCC. Where students did not complete one or both surveys following exit meetings, a follow-up survey sent 3 months after their formal exit was used for post-intervention scores. The specific tools in the survey were the Short Warwick–Edinburgh Mental Wellbeing scale (SWEMWBS) and the Process of Recovery Questionnaire (QPR). The SWEMWBS is a validated self-reported questionnaire used to measure well-being across both clinical and non-clinical populations. Scores range from 7 to 35, and support positively focused interventions, with items inquiring about the positive aspects of mental health.^23^

The QPR is a self-reported mental health recovery questionnaire, with a total score ranging from 0 to 60.^24^ The scale maps onto the CHIME framework (connectedness, hope and optimism, identity, meaning, empowerment), a conceptually defensible framework for personal recovery, developed through people’s experience.^25^ The psychometric properties and relevance of both survey tools are detailed in Appendix 2.

#### Education and employment outcomes

During enrolment and on formal exit from RCC, students were also asked about their current state of education and employment (five categories detailed in Table 3). Consent to use these data in the research study was sought.

#### NHS medical records data

Anonymous data related to use of specialist (secondary care) UK NHS mental health services were collected. Medical records were reviewed during RCC attendance, as well as the 6-month periods pre- and post-RCC intervention.

#### Description of changes

As part of the formal exit process and completion of exit documentation, students were also asked an open-ended question: ‘Since you have been involved with Recovery College Cornwall, please describe the biggest changes that have happened in your everyday life as a result of being involved in the project’. Answers were documented by students’ LSWs.

### Data analysis

#### Demographic data

Demographics were analysed with descriptive statistics.

#### Surveys

Survey results were paired (before and after intervention) with difference assessed for their fit to a normal distribution, using the Shapiro–Wilk test. All survey results were subjected to a paired Wilcoxon signed-rank test, and further testing with a paired *t*-test where normality applied. Statistical significance was shown with *P*-values. Paired sample *t*-tests also reported Hedges’ *g*-statistic as a measure of effect size, with a preset level of >0.8 used for determining clinically significant change. Pre- and post-scores for SWEMWBS were also compared with the SWEMWBS converted cut-off point for low well-being in the UK (19.5),^23^ to allow for analysis of proportion of individuals who moved from below to above this score following attendance at RCC.

#### Education and employment outcomes

Pre- and post-RCC intervention outcomes were compared. Students not engaged with education or employment (including employment searches) were classified as economically inactive. Where outcomes changed, students were required to both explicitly declare any change and provide appropriate documented or online evidence. These were educational course enrolment evidence, paid employment evidence, proof of registration and uploading of a CV online with a job search company and at least one job search.

#### NHS medical records data

Analysis focused on whether students were receiving specialist mental health support from NHS services during their time with RCC and the 6 months pre- and post-attendance at RCC. Referrals and discharge to and from services during these periods were also analysed.

#### Description of changes

A conventional content analysis^26^ to identify changes described by students on exiting RCC was undertaken. Answers provided by students were explicit, because of the focused question and recoding of responses, allowing for categorical themes to be generated through thematic analysis.^27^ This was undertaken by a single member of the research team. Another team member then separately scored a section of answers against these themes independently, to confirm consistency. Themes were compared quantitatively in relation to their occurrence.

## Results

### Demographics and data collected

A total of 154 students were approached to participate. 101 students consented to take part and 13 declined directly. Another 39 students declined indirectly through implied non-consent. This included not returning emails, telephone calls, text messages or consent forms after initially expressing an interest during online meetings. Demographic data collected is detailed for all (*n* = 101) consented students in Table 1. The majority of the student research participants (68%) were female, mean age was 38 (range 19–68, median 31, mode 24) years and all but one of the 92 out of 93 who responded to the question on ethnicity were White. Eighty-four students received the RCC educational intervention and were formally exited (agreed discharge/graduation). Seventeen students disengaged from RCC (and RCC input) before formal exit. The mean period of engagement for those graduating was 21 weeks (s.d. = 12.8). Fifteen students had face-to-face learning and telephone support, 24 had a combination of face-to-face and online learning along with telephone support, and the remaining 45 had online learning and telephone support (i.e. no face-to-face contact). Details of data collected and missing data (for study participants completing the interventions and study participants who disengaged) for each research measure are also detailed in Table 1.

**Table 1:** Demographics and data collected

Measure	Item	*n*
Gender	Male	33
	Female	68
Age at enrolment, years	<25	26
	25–35	21
	35–50	32
	>50	19
	Mean age	38
	s.d.	13.6
	No data:	
	Missing	3
Ethnicity	White British/English/Scottish/Welsh/Northern Irish	74
	White other	3
	White Cornish	15
	Dual heritage White and Black African	1
	No data:	
	Missing	8
Time with recovery college	8–15 weeks	36
	16–26 weeks	26
	27–40 weeks	10
	≥40 weeks	12
	Mean weeks	21
	Medium	18.5
	s.d.	12.8
	No data:	
	Disengaged students	17
Intervention received	Face-to-face learning and support	15
	Combination of face-to-face and online learning and support	24
	Online learning and support	45
	No data:	
	Disengaged students	17
SWEMWBS	Pre- and post-intervention scores total	Total 80 (100%)
	Recorded at exit	75 (87.5%)
	Recorded following exit (3 month)	5 (12.5%)
	No data:	
	Missing (students completing the intervention)	4
	Disengaged students	17
QPR	Pre- and post-intervention scores total	Total 75 (100%)
	Recorded at exit	54 (72%)
	Recorded following exit (3 months)	21 (28%)
	No data:	
	Missing (students completing the intervention)	9
	Disengaged students	17
Employment and education outcome data	Pre-and post-intervention	77
	No data:	
	Missing (students completing the intervention)	7
	Disengaged students	17
Medical records data	Students completing the intervention	77
	Disengaged students	15
	No data:	
	No permission (students completing the intervention)	7
	No permission (disengaged students)	2
Description of changes	Graduated students	68
	No data:	
	Missing (students completing the intervention)	16
	Disengaged students	17

SWEMWBS, Short Warwick–Edinburgh Mental Wellbeing Scale; QPR, Process of Recovery survey.

### Surveys

Pre- and post-intervention survey scores were available for 80 (out of 84) student research participants completing the RCC intervention for the SWEMWBS and 75 (of 84) for the QPR. Results are detailed in Table 2.

**Table 2:** Pre- and post-intervention statistical measures

	SWEMWBS	QPR
Mean scores pre-intervention (range)	17.3 (7.0–30.7)	27.2 (4.2–52.0)
Mean scores post-intervention (range)	21.9 (11.3–35.0)	38.8 (8.0–60.0)
Mean change score (changed from mean effect size)	8.6	8.8
Hedges’ *g*	1.08	1.03
Improvement with Wilcoxon signed-rank test	Yes (*P* < 0.001)	Yes (*P* < 0.001)
Improvement with paired *t*-test	Not applicable	Yes (*P* < 0.001)

SWEMWBS, Short Warwick–Edinburgh Mental Wellbeing Scale; QPR, Process of Recovery survey.

SWEMWBS scores across participants ranged from 7.0 to 30.7 before the intervention (mean 17.3). After the intervention, the mean increased to 21.9, within a range of 11.3–35.0.

Mean QPR scores changed from 27.2 pre- RCC intervention to 38.8 post-intervention, whereas the range changed across the intervention from 4.0–52.0 to 8.0–60.0 (Table 2).

There was evidence to suggest that differences in SWEMWBS scores before and after the RCC intervention did not follow a normal distribution (*P* < 0.001). This contrasted to the QPR scores, for which there was insufficient evidence to conclude that differences were not normally distributed. Differences in the QPR scores were subsequently further tested with a paired *t*-test.

A Wilcoxon signed-rank test demonstrated significant improvement in the SWEMWBS and QPR when pre- and post-scores were compared (both *P* < 0.001). In the case of QPR scoring, this positive conclusion was reinforced with additional testing using a paired *t*-test, which showed a significant improvement in scores (*P* < 0.001).

The mean increase in scores of 4.58 (s.d. = 4.76) for converted well-being (SWEMWBS) demonstrated a significant effect size (*t*(80) = 8.6, *P* < 0.01, Hedges’ *g* = 1.08). The mean change score for QPR was 8.8, which was also statistically significant (*P* < 0.01; Hedges’ *g* = 1.03).

Self-reported well-being and recovery scores were therefore found to increase significantly for student research participants who received the full intervention. At enrolment, 67 (83.75%) students who completed the SWEMWBS had scores indicative of what is defined as a low well-being score for the UK general population (>19.5).^28^ Of these 67 students, 46 (68.65%) moved from below to above this score following the RCC intervention.

### Education and employment outcomes

Education and employment outcomes are detailed in Table 3. Pre- and post-intervention data were available for 77 out of 84 student research participants who completed the intervention. More than half of students (43 of 68; 63%) who were unemployed or economically inactive when enrolling with RCC, were either in education, employment or have completed an active job search (following formal registration with an online employment company) at RCC exit. The number of students defined as economically inactive reduced from 53 (69%) at enrolment to 19 (24.4%) at the point of exit from RCC. Twelve (15.6%) of the students for whom data was available became formally employed (0 pre-intervention), and 25 had undertaken a job search. Only those in paid employment (ranging from office work to construction and seasonal holiday park positions) were classified as employed. Those registered in formal education or training doubled between pre- and post-intervention (from 9 to 18).

**Table 3:** Education and employment outcomes

Pre-intervention		Post-intervention (exit)	
Economically inactive	53 of 77 (68.8%)	Economically inactive	19 of 77 (24.4%)
Unemployed	15 (19.5%)	Unemployed	4 (5.1%)
In education or training	9 (11.7%)	In education or training	17 (23.1%)
Job search	0	Job search	25 (32.05%)
Employed	0	Employed	12 (15.4%)
Total	77	Total	77
	Missing: 7		Missing: 7
	Disengaged: 17		Disengaged: 17

Definitions: economically inactive, ‘without work or education and training and not seeking work’; unemployed, ‘without work, available for work and currently seeking work’; in education or training, ‘enrolled in education or training’; job search, ‘registration and uploading of a CV online with a job search company and at least one job search’; employed, ‘in paid employment’.

### Medical records data

Medical records reviewed for student research participants, including those who disengaged from RCC, are detailed in Table 4. Disengaged students who gave consent to review medical records were more likely to have current or recent records (9 out of 15; 60%) than those who remained with RCC for the duration of the intervention (28 out of 77; 36%).

**Table 4:** Secondary care mental health records data

	Students completing the intervention	Disengaged	All students
No records	35	3	38
Historical records	14	3	17
Current/recent records	28	9	37
Receiving support during RCC intervention	15	8	3
Discharged within 6 months before RCC enrolment	5	1	6
Discharge during RCC intervention	3	0	3
Discharged withing 6 months post-RCC intervention	2	0	2
Referred to services 6 months post-RCC intervention	3	0	3
No permission	7	2	9
Total	84	17	101

RCC, Recovery College Cornwall.

The 36% of RCC research participants completing the intervention who had recent or active secondary care mental health records (defined by mental health case-load) were either supported during the study (*n* = 20, 26%), 6 months pre-enrolment (*n* = 5, 6.5%) or 6 months post-intervention (*n* = 3. 4%). Five out of the 20 students who were supported when with RCC were either discharged during the study (*n* = 3) or 6 months post-exit (*n* = 2) from RCC. No students were referred for specialist mental health support when enrolled with RCC (either the 35 with no records or the five discharged less than 6 months before enrolment), but three were referred for support in the 6 months after the intervention.

### Qualitative data analysis of changes

Data were provided for 66 out of 84 students, with 115 changes described across three broad themes (Table 5). Two broad themes (‘within-self changes’ *n* = 57 and ‘practical changes’ *n* = 44) were described and had clear sub themes, whereas a third broad theme, ‘change from shared experience’, was also described explicitly by students (*n* = 14). All changes described were positive, with within-self changes around ‘confidence (self-esteem)’ described for half of the 66 responding students (*n* = 33). Practical changes related to skills learnt to improve or sustain mental health were identified by 24 students. Example quotes for themes are provided in Table 6.

**Table 5:** Description of what has changed

Within-self changes (*n* = 57)				Change from shared experience (*n* = 14)	Practical changes (*n* = 44)			
Confidence (self-esteem)	Outlook (positive/ enjoyment)	Insight	Other (calmness, control, meaning)	(Sharing and learning from others)	Skills/tools to maintain or improve mental health	Structure/focus	Working	Video/Skype skills
33	7	11	6	14	24	7	8	5

**Table 6:** Themes and example quotes

Broad themes	Subthemes	Examples
Within-self changes	Confidence (self-esteem)	Research participant 12 ‘I feel my confidence is up and my self-esteem. I am trying different things like exercising and a walking group. It's been fun being involved with a group. I feel more open to trying new things now’
	Outlook (positive/enjoyment)	Research participant 64 ‘The structure of the courses has allowed me to gain a better focus and the new material and tools I have gained have given me a positive belief about moving forward’
	Insight	Research participant 62 ‘I have experienced shifts in my self-awareness, meaning I felt more in tune with my needs on any given day, and can reframe accordingly’
Change from shared experience	Sharing and learning from others	Research participant 54 ‘Confidence to know that I could take part in these groups. It was good to know that everyone is in the same boat. Good to meet people and have discussion, and share experiences. I really valued the group experiences, it was nicer to be in a group than one to one’
Practical changes	Skills/tools to maintain or improve mental health	Research participant 94 ‘Being able to notice my negative thoughts and knowing that if I’m not able to deal with these straight away that is also ok, knowing that noticing is a step in the right direction. Being able to break things down into smaller chunks so I am able to process things more easily’
	Structure/focus	Research participant 82 ‘From starting at the Recovery college without direction, I found routine and structure’
	Working	Research participant 49 ‘I have started to volunteer in the XX charity shop in XX and am job searching for part time shop work within XX’

## Discussion

Data presented is for a large cohort of student research participants (*n* = 101) attending a single UK recovery college, of whom 84 completed the educational intervention and were exited (agreed discharge/graduation). This completion rate is higher than the 60–70% attendance rates reported at other colleges.^7^

Self-reported pre- and post-intervention well-being and recovery survey data, collected from a high percentage of student research participants, builds significantly on the growing but incomplete quantitative outcome evidence base evaluating the impact of recovery colleges on those who attend. Survey numbers (*n* = 80 SWEMWBS, *n* = 75 QPR) are larger than those published previously by recovery college studies replicating one or both of these self-reported survey methods when evaluating full interventions.^11,21,22^ Greater or similar differences in mean pre- and post-intervention scores are also evident. Statically significant changes are similar, but with higher student numbers and effect sizes than reported in the largest similar study using surveys, which reported *P* < 0.01 and effect sizes of 0.78 for the SWEMWBS and 0.75 for the QPR.^11^

Our effect sizes (1.08 for the SWEMWBS and for 1.03 the QPR) are higher than the preset level of >0.8 for determining clinically significant change. Despite limitations (discussed below), these data highlight the strength of our study findings and help contribute to a quantitative research evidence base that has been identified as limited.^4,13,29^

Pre- and post-intervention well-being scores (17.3 pre-intervention, 21.9 post-intervention) also indicate that mean SWEMWBS scores move from below to above the UK general population low well-being score (>19.5 for 15% of the UK population), after the RCC intervention. Any such comparison of uncontrolled independent populations requires caution, but at the individual level, minimal detectable change for the SWEMWBS is identified as 1–3 points,^23^ whereas other research indicates that this individual improvement of 1–3 points meets thresholds for statistically important change when people are seeking treatment for mental health problems.^28^

Changes in mean scores for the QPR study survey (27.2 to 38.8) are also higher than minimally important change reported for recovery for a single specific population of mental health patients.^30^

Economic activity outcomes detailed in our study also help address another specific area with limited evidence.^4^ Our findings arguably detail more explicit data on employment and educational activity for recovery college attendees than previously published.^10^ Formal employment for 12 (15.6%) of 84 graduating students, although modest, should be viewed in the context of the COVID-19 pandemic and its impact on job opportunities during this research study.^31^

Medical records data from our study does not quantify specific impact of RCC on service use, as reported from other recovery colleges,^18,19^ but does show that none of the RCC research participants were referred into specialist secondary care mental health services for additional support when receiving support from RCC.

The ‘changes’ reported by students following the RCC intervention can be viewed in light of the open survey and more in-depth qualitative focused research studies undertaken across other recovery colleges.^13^ Although focused on a single question, reports of positive change in ‘confidence (self-esteem)’ and ‘insight’ mirror descriptions of improvement in confidence and self-esteem,^9^ and self-awareness evidenced in other studies.^7,15^ Practical change in skills and tools for supporting mental health also correlate with the themes drawn from other studies.^13,15,32,33^ Citing of an explicit ‘change’ from having shared and learnt from peers arguably stems from the recovery college model of delivering interventions to groups simultaneously. The importance of working with peers, and the key role they play in improving students’ understanding and motivation, have been cited elsewhere.^14,17^ The enabling of different relationships (power, peers and working together) has also been recently identified as one of four key mechanisms of action at recovery colleges through a systematic review.^34^

### The impact of the COVID-19 pandemic on project delivery and its results

The COVID-19 outbreak forced the movement of RCC support and course delivery online early in this research study. Improvements are reported for a time period when pandemic restrictions were having a negative psychological impact.^35^ Results should also be considered in the context of an intervention that, despite being designed and originally implemented face to face with students, became a novel and untrialled form of recovery support, as it was fluidly developed and adapted for online delivery. Previous evidence has reported exclusively on face-to-face educational interventions. In our study, students continued to report positive changes in recovery and well-being despite the move to online learning and support. The changes in how mental health services and education are delivered post-pandemic is not yet fully determined, but recovery colleges in England and Ireland are already embracing a more permanent move to online recovery courses or forms of delivery.^36,37^ This study provides some early pragmatic evidence to support such delivery.

### Disengaging students

With 17 students (17%) disengaging from RCC, our data indicates that the RCC approach does not work for all. Five students anecdotally cited online learning as responsible when contacted during the COVID-19 pandemic, and this may have been a reason for others to disengage. The higher number of students who disengaged and were receiving specialist NHS mental health support (60%), compared with those who remained engaged with the college (36%), also indicates that those disengaging may have more complex mental health needs.

### Limitations

Not all students approached agreed to take part in this study. Thirteen explicitly declined (8.4% of students approached) and 39 (25% of students approached) implied non-consent following COVID-19 restrictions from March 2020 and the need for potential participants to provide additional online consent to contact, attend online researcher meetings and return consent forms in the post. The complex information governance process and additional consent requirements may have influenced these students not providing consent, but data were not collected to ask why students did not consent to the study. Data were also not collected to ask why students disengaged from RCC or to characterise either population in terms of mental health diagnosis or other factors.

The COVID-19 pandemic affected students’ recovery journeys. A number remained on hold (not attending courses) for a period of time before formally re-engaging once familiar with video conferencing. This affected the mean length of time with RCC, with a significant varied length of time spent with RCC (mean 21 weeks, s.d. = 12.8) resulting in different intensities of RCC intervention. Survey data was also collected 3 months post-attendance for some students (SWEMWBS: 5 out of 80, 12%; QPR: 21 out of 75; 28%), meaning not all surveys were completed at the same time. This may have also affected the role of RCC intervention in well-being and recovery scores.

Ethnicity in particular, although mirroring the local population,^38^ is not diverse. In addition, although varying restrictions to referral is not uncommon,^9^ employment status is not cited elsewhere as a restricting factor for recovery college attendance at other colleges.

There is no control group for this study, limiting potential to infer causality. Other confounding variables may have also impacted, including the COVID-19 pandemic or spontaneous recovery. One in four (26%) research participants who completed the intervention (and gave consent to access to secondary care mental health records) were also on a specialist NHS mental health case-load at some point during their time as a student with RCC. They were therefore supported in some capacity by the NHS in addition to RCC. As cited elsewhere, individuals who self-refer to a recovery college may also be ready to ‘recover’, an important factor to consider when comparing to other populations. The personal, subjective, complex and often non-lineal process of recovery were also not researched in this study.

In conclusion, this study adds some quantitative research findings to the building evidence base regarding the impact of recovery colleges, and specifically, recovery college interventions that include remote delivery. This adds to the debate around the role of recovery colleges and the recovery college model as both a complementary and alternative intervention to traditional mental health services. Causality cannot be inferred because of the lack of control group, but findings arguably add to the calls for such robust evidence through randomised controlled trials.^4,13,29^

Randomised controlled trials for mental health care are costly, time-consuming and face complex ethical and methodological considerations.^39^ These are arguably complicated by the varied structures of recovery colleges and fidelity to the original model.^14^ Our study does not explicitly discuss findings in relation to RCC and the fidelity of other recovery colleges to this model, but do show evidence that a recovery college can be flexible and adaptable in relation to how interventions are delivered. Since our study data were collected, the rationale and protocol for a multifaceted 5-year programme formally characterising and testing recovery colleges has also been published.^40^ This includes in-depth qualitative research, designed to understand generalisability of findings along with the role of co-production. This programme of work may not only significantly build outcome evidence data, but also raise understanding as to why recovery colleges evolve as they do and why they have become more established in certain countries such as the UK, Australia and Canada, but have struggled to gain a foothold elsewhere, such as the USA.

## Data Availability

The data that support the findings of this study are available from the corresponding author, R.S., upon reasonable request.

## Appendix 1. Recovery College Cornwall, UK

From its opening in late 2019 and before completion of this study, Recovery College Cornwall (RCC) was an innovative 3-year European Social Fund (ESF) pilot project, with enrolment open to anyone with mental ill health, aged over 18 years and unemployed (unemployment being a specific ESF funding stipulation). The student research population in this paper indicate that approximately 41.5% of attendees will not have received support from specialist mental health services when enrolling, 18.5% will have had historical (more than 6 months pre-enrolment) support and 40% will have recent (within 6 months of enrolment) or current specialist support.

The college was developed in line with the UK recovery college model, and is consistent with recent fidelity measures.^7,9^ It focuses on delivering courses to support recovery from mental ill health through learning, as well as encouraging individuals to be the agents of their own recovery and empowering them to live the life they choose. Students self-refer. Co-production principles are embedded, with courses shaped and delivered by people with lived experience of mental ill health, alongside educational and mental health professionals.

Intervention courses are focused on helping students build their understanding and management of mental health, well-being, motivation and resilience. There are also vocational components of specific courses offering the opportunity to develop practical skills relating to engagement, education, training and employment. Students may engage in different ways, ranging from attendance at a small number of courses to working toward becoming a RCC peer mentor. Participation is therefore personalised and dependent on specific requirements and needs.

For the first 8 weeks of this study, courses were delivered face to face (to up to ten learners) and across Cornwall. Following a pause during the second week of March 2020 onward and instruction for the UK population to stay at home and socially distance,^41^ courses were almost exclusively delivered online and via Skype (up to five learners). An unpublished RCC audit undertaken at the time of this study indicated that an average of between four and five courses are attended.

Two formal trainers with significant experience working in the education and community sectors were funded to work at RCC for the duration of the ESP funding period. Six learning support workers (LSWs) supported students and the delivery of courses. Four were from a Cornish charity that provides support and guidance to people recovering from mental ill health and had received mental health training. The other two were seconded from Cornwall Partnership NHS Foundation Trust (CPFT) and had worked in clinical support and nursing roles for mental health patients at CPFT.

All students were also allocated an LSW. LSWs support enrolment processes and provide direction and reflection around courses. They also provide support to embed learning and progression toward recovery goals and outcomes, and support regarding education, training and employment goals. Tailored and practical personalised support is also provided, including health advice, safeguarding and a variety of other issues ranging from IT support to administrative tasks. Audit data suggests that students at RCC receive an average of 5 h personalised one-to-one support from their LSWs.

## Appendix 2. Evidence base for surveys used

The Short Warwick–Edinburgh Mental Wellbeing Scale (SWEMWBS) has been applied across recovery college evaluations,^10^ and has the capacity to detect change and subtle improvements in populations with both good and poor mental health.^42^ Respondents answer seven questions in the SWEMWBS, which are all scored on a five-point Likert scale (1–5). A score conversion method for raw scores is applied to allow for comparison with results from other studies. A cut-off point for low well-being (19.5) has been identified through analysis of general UK population data (bottom 15% of the population).^23^ A recent benchmarking study suggests converted scores of >18–20 are indicative of possible mild depression.^29^ Although not designed to clinically monitor individual recovery, the SWEMWBS have been shown to be responsive to change at the individual level, with a minimum of one point and a maximum of three seen as having some clinical significance.^24^

The psychometric properties of the Process of Recovery Questionnaire (QPR) support its use in clinical practice and recovery services.^43^ It has also been used to evaluate recovery colleges.^11,22^ Respondents answer 15 questions on a five-point Likert scale (0–4). The minimal important difference for the QPR is not identified, but a within-person difference of five (individual level) and four (between-group) have been suggested for recovery from a specific mental health condition.^30^
